# The influence of partial gastrectomy for gastric cancer on the spontaneous disappearance of *Helicobacter pylori*: A single‐center prospective study

**DOI:** 10.1002/cnr2.1903

**Published:** 2023-09-12

**Authors:** Senichiro Yanagawa, Nobuaki Fujikuni, Kazuaki Tanabe, Masahiro Nakahara, Toshio Noriyuki

**Affiliations:** ^1^ Department of Surgery Onomichi General Hospital Hiroshima Japan; ^2^ Department of Digestive Surgery Hiroshima Prefectural Hospital Hiroshima Japan; ^3^ Department of Perioperative and Critical Care Management, Graduate School of Biomedical and Health Sciences Hiroshima University Hiroshima Japan

**Keywords:** gastric cancer, *Helicobacter pylori*, partial gastrectomy, spontaneous disappearance

## Abstract

**Background/Aims:**

*Helicobacter pylori* (HP) eradication is recommended after endoscopic treatment of early gastric cancer (EGC). Cases of spontaneous HP resolution after partial gastrectomy due to environmental changes have been reported; however, there is no evidence for the efficacy of HP eradication in suppressing carcinogenesis and also no reports on the natural history of HP after partial gastrectomy in gastric cancer (GC). To report the natural history of HP in patients with GC and HP infection after partial gastrectomy.

**Methods and Results:**

We prospectively studied the rate of spontaneous disappearance of HP after partial gastrectomy in patients with GC. From April 2016 to May 2020, 80 patients underwent partial gastrectomy, including 9 cases of proximal gastrectomy (PG), and 71 cases of distal gastrectomy (DG). The presence of HP was confirmed in the stool antigen test 1 year after operation, HP infection persisted in 46 patients (57.5%) and disappeared in 34 patients (42.5%). In univariate analysis, only proton pump inhibitor (PPI) use was a significant contributing factor for the spontaneous resolution of HP infection, especially in the DG group. However, there was no difference in the rates of HP disappearance between Billroth‐I and Roux‐en‐Y reconstructions in the DG group.

**Conclusion:**

The HP spontaneously disappeared in 42.5% of the GC patients within 1 year after partial gastrectomy. Further investigation in a larger cohort is needed to elucidate the underlying mechanisms.

## 1 INTRODUCTION

Gastric cancer (GC), which occurs in approximately in 1 million new patients each year, is the third leading cause of cancer‐related deaths worldwide especially in Asia.^1^ GC is strongly associated with *Helicobacter pylori* (HP) infection. The Japanese Society for Helicobacter Research first published the “Guidelines for the Management of *Helicobacter pylori* infection in Japan” in 2000 and revised the guidelines in 2019.^2^ In addition, HP is a major cause of gastric carcinogenesis and is associated with an increased risk of chronic gastritis, atrophic gastritis, peptic ulcer disease, intestinal metaplasia, dysplasia, and mucosa‐associated lymphoid tissue (MALT) lymphoma. The mechanism of HP‐induced GC includes HP‐induced atrophic gastritis and intestinal metaplasia, epithelial‐mesenchymal transition, and the generation of GC stem cells.^3^, ^4^ AS for GC treatment, early GC (EGC) with very low risk of lymph node metastasis are often treatable by endoscopic mucosal resection (EMR) or endoscopic submucosal dissection (ESD).^1^ GC that cannot be cured by EMR or ESD should be treated by partial or total gastrectomy with lymph node dissection.^1^ Moreover, postoperative adjuvant chemotherapy is recommended for patients with pStgaeIIor more advanced GC (AGC),^5^, ^6^ and the efficacy of neoadjuvant chemotherapy for locally advanced resectable GC has also been reported.^1^, ^7^ Therefore, eradication of HP is necessary to prevent GC. A series of randomized control and cohort studies have suggested the value of HP eradication for the prevention of GC, MALT lymphoma, and other benign diseases.^8^ It is widely accepted that HP eradication reduces tumor recurrence, metachronous cancers and improves survival in patients undergoing endoscopic resection for EGC.^4^, ^8^, ^9^, ^10^ Therefore, HP eradication is widely recommended in most guidelines for patients undergoing endoscopic resection for EGC.

HP eradication after partial gastrectomy, including in the cases of EGC and AGC, has been previously reported.^11^, ^12^ In a prospective randomized HP eradication trial of 169 patients after partial gastrectomy, there was no statistically significant difference in long‐term survival.^11^ Therefore, HP eradication may not always be beneficial for patients. However, there have been no reports on the natural history of HP after partial gastrectomy.

This study aimed to observe the natural history of HP in GC patients with HP infection after partial gastrectomy.

## 2 MATERIALS AND METHODS

### 2.1 Objective

This study aimed to prospectively observe the rate of spontaneous disappearance of HP after partial gastrectomy for GC. The clinical and pathological data on patient's information were extracted from the medical records. The HP infection status was assessed preoperatively and 1 year after postoperatively. For patients who were positive for HP infection at 1 year postoperative, HP infection status was also assessed at 3 years postoperative if desired by the patient. Patients younger than 20 years were excluded.

### 2.2 Patients

**FIGURE 1:**
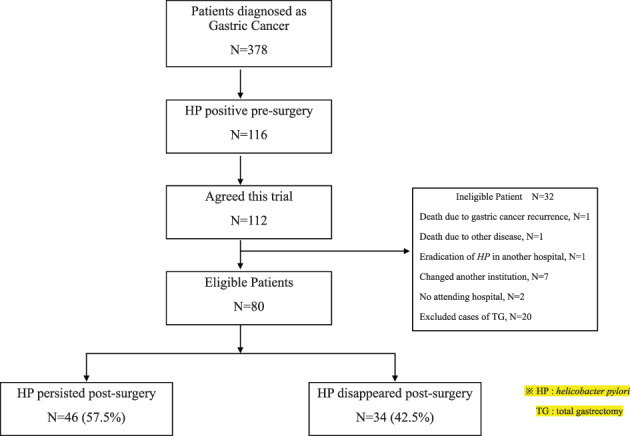
CONSORT diagram for the trial.

Using previous literature, clinical and pathological data that may be related to the development of GC and HP were collected from the patients' medical records. Between April 2016 and May 2020, 378 patients were diagnosed with GC using esophagogastroduodenoscopy in accordance with the Union for International Cancer Control (UICC) tumor node metastasis (TNM) classification and underwent surgical treatment in our hospital. Before surgery, 116 patients were positive for HP and 185 were negative and 66 were not examined, and 11 patients received eradication of HP. Among the 116 patients who were HP‐positive, 112 patients agreed to participate in this prospective trial, wherein they had no eradication of HP after surgery and were retested for the presence of HP antigen in the stool after 1 year. Of these, 20 patients underwent total gastrectomy (TG), 1 patient died of GC, 1 patient died of a different disease, 1 patient received eradication of HP at another institution, 7 patients were transferred to another institution, and 2 patients were lost to follow up at our hospital. These 32 patients were excluded from the trial ultimately, 80 patients were included (Figure 1).

This trial was retrospectively registered in the University Hospital Medical Information Network (UMIN) Clinical Trials Registry (UMIN ID: UMIN000046614; Registration date: 12/01/2022).

### 2.3 Surgical procedure

**FIGURE 2:**
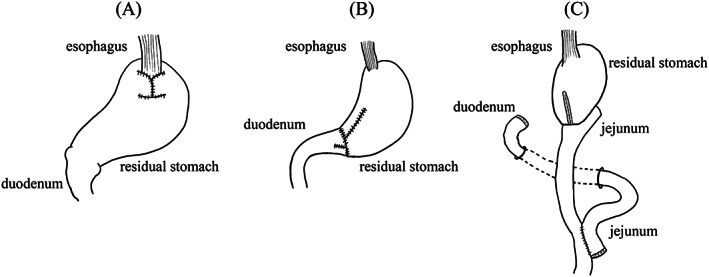
(A) Proximal gastrectomy with the double flap technique: Esophagus and residual stomach are anastomosed. (B) Distal gastrectomy with Billroth‐I reconstruction: Residual stomach and duodenum are anastomosed. (C) Distal gastrectomy with Roux‐en‐Y reconstruction: Residual stomach and jejunum are anastomosed. In addition, jejunum and jejunum are anastomosed.

We performed proximal gastrectomy (PG) using the double flap technique (Figure 2A) in the Fundus region of GC and distal gastrectomy (DG) using Billroth‐I (B‐I) (Figure 2B) or Roux‐en‐Y (RY) (Figure 2C) reconstruction in the Corpus or Antrum and Pylorus region of GC.^13^, ^14^ In cases of DG, we selected B‐I reconstruction whenever possible. We did not perform Billroth‐II (B‐II) reconstruction.

In all cases, preoperative imaging was used to identify the tumor location and progression. Accordingly, appropriate partial gastrectomy selection and lymph node dissection were performed to avoid TG as much as possible.

### 2.4 HP status evaluation

The stool antigen test (SAT) is a noninvasive method for the diagnosis of HP infection, with a good sensitivity and specificity of 94% and 97%, respectively.^15^ All eligible patients underwent pre‐ and post‐operative HP SATs using a monoclonal antibody‐based enzyme immunoassay (EIA) using the TESTMATE Pylori Antigen EIA^R^ (Catalog No. 4987‐295‐48705‐0, Wakamoto Pharmaceutical Co., Japan).

### 2.5 Statistical analysis

All statistical analyses were performed using EZR (Saitama Medical Centre, Jichi Medical University, Saitama, Japan), a graphic user interface for R (The R Foundation for Statistical Computing, Vienna, Austria). More precisely, it is a modified version of the R commander designed to add statistical functions frequently used in biostatistics.^16^

Student's *t*‐test and a Chi‐squared test were used to compare baseline characteristics between the HP‐persisted and HP‐disappeared groups (Tables 1 and 3). Comparisons between the HP‐persisted and HP‐disappeared groups were made using the Fisher's exact test, because there was a group with less than 40 cases (Table 2). Statistical significance was set at *p* < .05.

**TABLE 1:** Patient characteristics and comparison of *Helicobacter pylori*‐persisted and ‐disappeared groups after gastrectomy.

Parameter	Total *N* = 80	HP persisted *N* = 46	HP disappeared *N* = 34	*p*‐value
Age (mean ± SD)	69	68 ± 11	69 ± 11	.624
Body mass index (mean ± SD)	23	23.1 ± 5.0	22.7 ± 3.4	.611
Sex				1
Male	56	32	24	
Female	24	14	10	
Surgery type				.991
PG	9	5	4	
DG with B‐I	31	18	13	
DG with RY	40	23	17	
Surgery approach				.55
Open	13	6	7	
Laparoscopic	67	40	27	
Tumor location				.166
Fundus	9	5	4	
Corpus	51	33	18	
Antrum and pylorus	20	8	12	
pT				.827
1	47	28	19	
2–4	33	18	15	
pN				.173
0	57	36	21	
1–3	23	10	13	
M				.726
0	75	44	31	
1	5	2	3	
pStage				.306
I	51	32	19	
II, III, IV	29	14	15	
Histopathology				.422
Intestinal type	39	21	18	
Diffuse type	39	23	16	
Indeterminate type	2	2	0	
Chemotherapy after operation				.208
Yes	17	7	10	
No	63	39	24	
Smoking history				.313
Yes	43	22	21	
No	37	24	13	
Alcohol history				.578
Yes	37	23	14	
No	43	23	20	
Atrophic gastritis				.484
Positive	54	33	21	
Negative	26	13	13	
PPI medication				**.0292**
Yes	28	11	17	
No	52	35	17	
Antibiotics medication				.36
Yes	20	13	7	
No	60	33	27	

*Note*: *p*‐value was calculated using Student *t*‐test and Chi‐square test for categorical variables. Bold indicates that the *p*‐value is 0.05 or less and that the difference is statistically significant.

Abbreviations: B‐I, Billroth‐I; DG, distal gastrectomy; HP, *Helicobacter pylori*; PG, proximal gastrectomy; PPI, proton pump inhibitor; RY, Roux‐en‐Y; SD, standard deviation.

**TABLE 2:** (A) Relationship between *Helicobacter pylori* infection and proton pump inhibitor use in the proximal gastrectomy group. (B) Relationship between *Helicobacter pylori* infection and proton pump inhibitor use in the distal gastrectomy group. (C) Relationship between *Helicobacter pylori* infection and proton pump inhibitor use in the distal gastrectomy with Billroth‐I reconstruction group. (D) Relationship between *Helicobacter pylori* infection and proton pump inhibitor use in the distal gastrectomy with Roux‐en‐Y reconstruction group.

(A) PG group				
PPI use	Total *N* = 9	HP persisted *N* = 5	HP disappeared *N* = 4	*p*‐value
Yes	7	3	4	.444
No	2	2	0	

(B) DG group				
PPI use	Total *N* = 71	HP persisted *N* = 41	HP disappeared *N* = 30	*p*‐value
Yes	21	8	13	**.0377**
No	50	33	17	

(C) DG with B‐I group				
PPI use	Total *N* = 31	HP persisted *N* = 18	HP disappeared *N* = 13	*p*‐value
Yes	10	4	6	.247
No	21	14	7	

(D) DG with RY group				
PPI use	Total *N* = 40	HP persisted *N* = 23	HP disappeared *N* = 17	*p*‐value
Yes	11	4	7	.153
No	29	19	10	

*Note*: *p*‐values were calculated using Fisher's exact test for categorical variables. Bold indicates that the *p*‐value is 0.05 or less and that the difference is statistically significant.

Abbreviations: B‐I, Billroth‐I; DG, distal gastrectomy; HP, *Helicobacter pylori*; PG, proximal gastrectomy; PPI, proton pump inhibitor; RY, Roux‐en‐Y.

## 3 RESULTS

A total of 378 patients were diagnosed with GC according to the UICC TNM classification at our hospital during the study period. Data from 80 patients were collected according to the CONSORT diagram for this prospective trial (Figure 1).

After the first postoperative year, 46 patients tested positive for HP infection (57.5%) and HP had disappeared in 34 patients (42.5%). Among the 46 patients who tested positive 1 year after partial gastrectomy, 11 were also tested 3 years postoperatively, and 3 tested negative for HP without eradication (27.3%). Patient characteristics and results of the univariate analysis are shown in Table 1. There were no significant differences in age, body mass index (BMI), sex, surgical approach, tumor location, pathological tumor depth (pT), pathological lymph node metastasis (pN), distant metastasis (M), histological characteristics of the tumor, chemotherapy after operation, history of smoking, history of alcohol consumption, presence of preoperative atrophic gastritis, or, antibiotic use during the first year after surgery (treatment other than HP eradication). In contrast, there was a statistically significant difference in proton pump inhibitor (PPI) use during the first year after surgery between the two groups (*p* = .0292) (Table 1). Regarding the history of PPI use after partial gastrectomy, 9 patients used PPI for 4 weeks, 6 patients for 8 weeks, 1 patient for 12 weeks, 1 patient for 16 weeks, 1 patient for 24 weeks, 2 patient for 32 weeks, 1 patient for 36 weeks, and 7 patients for 40 weeks. The oral dose of PPIs was 10 mg/day in 10 patients and 20 mg/day in 18 patients.

Regarding the disappearance of HP, postoperative PPI use showed no significant difference among PG cases (*p* = .444) (Table 2A), but showed a significant difference among DG cases (*p* = .0377) (Table 2B). Furthermore, among the DG cases, there was no significant difference in PPI use between those who underwent B‐I and RY reconstruction (*p* = .247, .153) (Table 2C,D).

A comparison of surgical procedures showed no statistically significant difference in the spontaneous resolution rate of HP between PG and DG (*p* = 1) (Table 3A). Among the patients who underwent DG, there was no statistically significant difference in surgical procedures between those who underwent B‐I and RY reconstruction (*p* = 1) (Table 3B).

**TABLE 3:** (A) Comparison between proximal gastrectomy and distal gastrectomy. (B) Comparison between distal gastrectomy with Billroth‐I and Roux‐en‐Y reconstructions.

Surgery type	Total *N* = 80	HP persisted *N* = 46	HP disappeared *N* = 34	*p*‐value
(A)				
PG	9	5	4	1
DG	71	41	30	

Surgery type	Total *N* = 71	HP persisted *N* = 41	HP disappeared *N* = 30	*p*‐value
(B)				
DG with B‐I	31	18	13	1
DG with RY	40	23	17	

*Note*: *p*‐values were calculated using Chi‐square test for categorical variables.

Abbreviations: B‐I, Billroth‐I; DG, distal gastrectomy; HP, *Helicobacter pylori*; PG, proximal gastrectomy; RY, Roux‐en‐Y.

## 4 DISCUSSION

Screening and eradication of HP are recommended in many guidelines.^17^ A randomized controlled study of EGC patients undergoing endoscopic resection reported that HP eradication reduces the incidence of metachronous GC or gastric neoplasia.^4^, ^8^, ^9^, ^10^ However, a prospective study of HP eradication after partial gastrectomy revealed that atrophic gastritis and intestinal metaplasia were associated with the spontaneous disappearance of HP.^18^ Some retrospective studies have reported that HP eradication after partial gastrectomy influences the long‐term prognosis, but no consensus has been reached.^9^, ^10^, ^11^, ^12^, ^19^ In addition, previous reports have not considered the possibility of spontaneous HP disappearance after partial gastrectomy.

Thus, we considered the possibility of spontaneous disappearance of HP, and prospectively studied patients with preoperative HP infection who did not undergo postoperative HP eradication. Of the 80 patients in this trial, 46 tested positive (57.5%), and 34 tested negative (42.5%) for HP infection at 1 year after surgery. In univariate analysis, only PPI use was a statistically significant factor (Table 1). We examined whether surgical techniques and PPI use had an effect on the spontaneous disappearance of HP and observed a statistically significant difference among DG cases (Table 2B). As noted in the Section 3, 28 patients had been taking PPI postoperatively for 4–40 weeks. To maintain a sensitivity of 94% and specificity of 97% for the HP stool antigen test, an interval of at least 4 weeks after HP eradication is necessary.^18^ Therefore, the probability of a false‐negative result due to PPI use was extremely low because there was no oral PPI period of more than 4 weeks prior to the test.

In general, changes in postoperative HP infection status have been suggested to be related to bile reflux and dramatic changes in acid secretion after partial gastrectomy, which appear to inhibit HP growth in the remnant stomach.^20^ A previous study reported that the spontaneous clearance rate of HP is related to the type of surgery performed. B‐II reconstruction has a higher bile reflux rate than B‐I reconstruction^3^, ^21^; therefore, RY reconstruction is preferable when B‐I reconstruction is difficult. There was a tendency for more bile reflux in B‐I reconstruction and a smaller remnant stomach in RY reconstruction, and we investigated how this might be related to the spontaneous disappearance of HP. However, the results showed no statistical difference between B‐I and RY reconstructions in the rate of spontaneous clearance of HP (Table 3A,B). The double flap technique was performed in 9 patients with PG, and spontaneous resolution of HP was observed in 4 patients. To the best of our knowledge, there is no evidence of an association between PG and HP status and this is the first report to compare B‐I and RY reconstruction in terms of spontaneous disappearance of HP. Therefore, as reported by Basso et al.,^20^ the inclination toward alkalinity due to bile reflux and the replacement of the residual gastric mucosa with an infection‐resistant mucosa after partial gastrectomy may lead to decreased incidence of HP infection. It is also conceivable that postoperative PPI use may have further suppressed acid secretion and contributed to the spontaneous disappearance of HP. However, as noted in Section 3, only 3 of 11 cases (27.3%) examined after 3 years demonstrated spontaneous disappearance of HP. Therefore, patients with positive HP status 1 year after partial gastrectomy also have a high rate of positivity 3 years after partial gastrectomy, suggesting that if the HP does not disappear spontaneously in the first postoperative year, the rate of spontaneous HP disappearance is low even in the long term.

Recently, it has been reported that chemotherapy for non‐hematological malignancies without prophylactic antibiotic administration affects the reduction or composition of the gut microbiota during myelosuppression.^22^ It has long been known that chemotherapy inhibits bacterial growth, and Maier et al.^23^ have demonstrated in vitro that chemotherapy affects a wide range of commensal bacteria. We found no statistically significant difference in HP disappearance between patients who did and did not receive postoperative chemotherapy, but the rate of HP disappearance was higher in the postoperative chemotherapy group (Table 1), suggesting that chemotherapy for GC may have influenced HP disappearance. Larger trials are warranted to confirm the existence of a statistically significant difference.

This trial had some limitations, including its single‐center design and the relatively small number of patients. Although we were not able to elucidate the mechanism of the spontaneous disappearance of HP, we believe that the results are valuable, as this is a rare prospective investigation of the natural history of HP infection status in patients who underwent partial gastrectomy for GC without HP eradication.

## 5 CONCLUSION

Spontaneous HP resolution was observed 1 year postoperatively in 42.5% of patients with GC. We considered acid suppression by environmental residual stomach and postoperative PPI use or changes in intestinal microbiota due to chemotherapy as possible reasons for the spontaneous resolution; however, the exact mechanism remains unclear. This study suggests that a mechanism for spontaneous disappearance of HP may exist. If a larger cohort study can yield adequate reproducibility regarding the results of rate of spontaneous HP disappearance, the mechanism of said spontaneous disappearance can be elucidated, which in turn will help to avoid unnecessary HP eradication and reduce the incidence of HP‐related GC.

## AUTHOR CONTRIBUTIONS

**Senichiro Yanagawa:** Conceptualization (lead); data curation (lead); formal analysis (lead); investigation (lead); methodology (lead); resources (lead); visualization (lead); writing – original draft (lead); writing – review and editing (lead). **Nobuaki Fujikuni:** Conceptualization (equal); investigation (equal); methodology (equal); project administration (lead); writing – review and editing (equal). **Kazuaki Tanabe:** Data curation (equal); writing – review and editing (supporting). **Masahiro Nakahara:** Resources (equal); writing – review and editing (supporting). **Toshio Noriyuki:** Resources (equal); supervision (lead); writing – review and editing (supporting).

## CONFLICT OF INTEREST STATEMENT

The authors have stated explicitly that there are no conflicts of interest in connection with this article.

## ETHICS STATEMENT

This study was conducted in accordance with the Declaration of Helsinki, approved by the Institutional Review Board of Onomichi General Hospital ethics review committee (OJH‐202156), and retropectively registered in the UMIN Clinical Trials Registry (UMIN ID: 000046614; registration date: 12/01/2022).

## INFORMED CONSENT STATEMENT

Informed consent was obtained from all subjects involved in the study.

## Data Availability

The datasets used and analyzed during the current study are available from the corresponding author upon reasonable request.
